# Flavonoid Intake in European Adults (18 to 64 Years)

**DOI:** 10.1371/journal.pone.0128132

**Published:** 2015-05-26

**Authors:** Anna Vogiatzoglou, Angela A. Mulligan, Marleen A. H. Lentjes, Robert N. Luben, Jeremy P. E. Spencer, Hagen Schroeter, Kay-Tee Khaw, Gunter G. C. Kuhnle

**Affiliations:** 1 Department of Food & Nutritional Sciences, University of Reading, Reading, United Kingdom; 2 Department of Public Health and Primary Care, University of Cambridge, Cambridge, United Kingdom; 3 Mars, Inc., McLean, VA, 22101, United States of America; 4 University of Cambridge, School of Clinical Medicine, Clinical Gerontology Unit, Cambridge, United Kingdom; Harbin Medical University, CHINA

## Abstract

**Background:**

Flavonoids are a group of phenolic secondary plant metabolites that are ubiquitous in plant-based diets. Data from anthropological, observational and intervention studies have shown that many flavonoids are bioactive. For this reason, there is an increasing interest in investigating the potential health effects of these compounds. The translation of these findings into the context of the health of the general public requires detailed information on habitual dietary intake. However, only limited data are currently available for European populations.

**Objective:**

The objective of this study is to determine the habitual intake and main sources of anthocyanidins, flavanols, flavanones, flavones, flavonols, proanthocyanidins, theaflavins and thearubigins in the European Union.

**Design:**

We use food consumption data from the European Food Safety Authority (EFSA) and the FLAVIOLA Food Composition Database to estimate intake of flavonoids.

**Results:**

Mean (±SEM) intake of total flavonoids in Europe was 428±49 mg/d, of which 136±14 mg/d were monomeric compounds. Gallated flavan-3-ols (53±12 mg/d) were the main contributor. The lowest flavonoid intake was observed in Mediterranean countries (monomeric compounds: 95±11 mg/d). The distribution of intake was skewed in many countries, especially in Germany (monomeric flavonoids; mean intake: 181 mg/d; median intake: 3 mg/d).

**Conclusions:**

The habitual intake of flavonoids in Europe is below the amounts found to have a significant health effect.

## Introduction

Flavonoids are a group of secondary plant metabolites, derived from 2-phenylchroman, which are ubiquitous in seed plants (spermatophytes) and therefore found in most plant-based foods [1]. They exist both in monomeric (e.g. flavanols or anthocyanidins), and in polymeric form (e.g. proanthocyanidins, theaflavins and thearubigins). Flavonoids have a wide range of biological function in plants [2], such as the protection from UV radiation and as signalling molecules for interaction with microbes [3]. Flavonoid-containing plants have also a long history of medicinal use: for example the catechin-monomer rich extract of *Acacia*, *catechu*, has been described as astringent in European pharmacopoeias since almost 400 years [4], and has also been recommended to treat heart diseases [5]. In the 20^th^ century, flavonoids were briefly considered to be a vitamin affecting capillary permeability [6], although it emerged quickly that these compounds are not an essential dietary factor [7]. However, based on these observations, flavonoid-containing drugs have been used extensively not only to treat disorders of the peripheral circulation, but also to treat radiation poisoning, intoxications and liver diseases [8].

In recent decades, there has been increasing interest in the potential beneficial effect of habitual flavonoid consumption on human health. Data from anthropological research [9] and observational studies [10, 11] suggested that they can reduce the risk of cardio-vascular diseases (CVD) although results from more recent studies have been less clear [12–15]. Due to their ability to scavenge free radicals *in vitro* they have often been described as *antioxidants* although it has been shown that this is an unlikely explanation for their activity *in vivo* [16]. A recent meta-analysis of observational epidemiological studies suggested an overall beneficial effect of flavonoid intake [17], however, the data available are more ambiguous. McCullough *et al*. find the main beneficial effect emerge when comparing very low intake with low intake [13] and this can be observed in other studies [12, 14]. While this could be explained by a threshold effect, there are also alternative explanations such as differences in dietary composition [15] or other unknown confounding factors. It is therefore not possible to attribute the observed effect to flavonoid intake alone. There are also no consistent associations between flavonoid intake and cancer risk. A recent meta-analysis showed no beneficial association between flavonoid intake and breast cancer risk [18] or gastric cancers [19], while there was a beneficial effect with smoking-related lung cancer [20] although only in some populations [21]. As with cardiovascular diseases, the main beneficial effect is often found when comparing very low with low intakes [22]. This is in marked contrast to results from a recent study investigating associations between flavonoid intake and the risk of type 2 diabetes in the EPIC cohort [23]: in this study, higher intake is associated with lower risk at least for some compounds, and the analyses show statistically significant trends.

While considerable attention has been focused on the beneficial health effects of flavonoids, excessive intake can also result in significant adverse effects [24], however there is a paucity of data on safety. Adverse affects range from mild gastro-intestinal symptoms to severe conditions such as haemolytic anaemia. Although flavonoids have been used as hepatoprotective drugs, flavonoids—and in particular gallated compounds found in green tea [25]—have been associated with increased risk of hepatotoxicity [24]. Flavonoid consumption has also been linked to haemolytic anaemia, and while this is best documented for (+)-catechin containing drugs [8], it is documented for other flavonoids as well [26, 27]. Flavonoids can also interact with cell proliferation by inhibiting topoisomerase 2 [28] and this can have both, a pro- and an anti-carcinogenic effect; some epidemiological data suggest an increased risk of leukaemia in neonates following high intake of bioflavonoids by mothers [29, 30]. Furthermore, the extensive hepatic metabolism of flavonoids can result in extensive flavonoid-drug interactions, especially as flavonoids can induce and inhibit cytochrome P450 enzymes [31].

There is an increased interest in exploring the benefits of establishing dietary recommendation for *bioactives* such as flavonoids [32], but there are still insufficient data on efficacy and safety for most compounds. Although dietary intervention studies have been conducted to investigate the beneficial effect of flavonoids on health, most notably with flavan-3-ols [33], the lack of standardised protocols and well defined intervention vehicles makes it often difficult to interpret the results.

**Fig 1:**
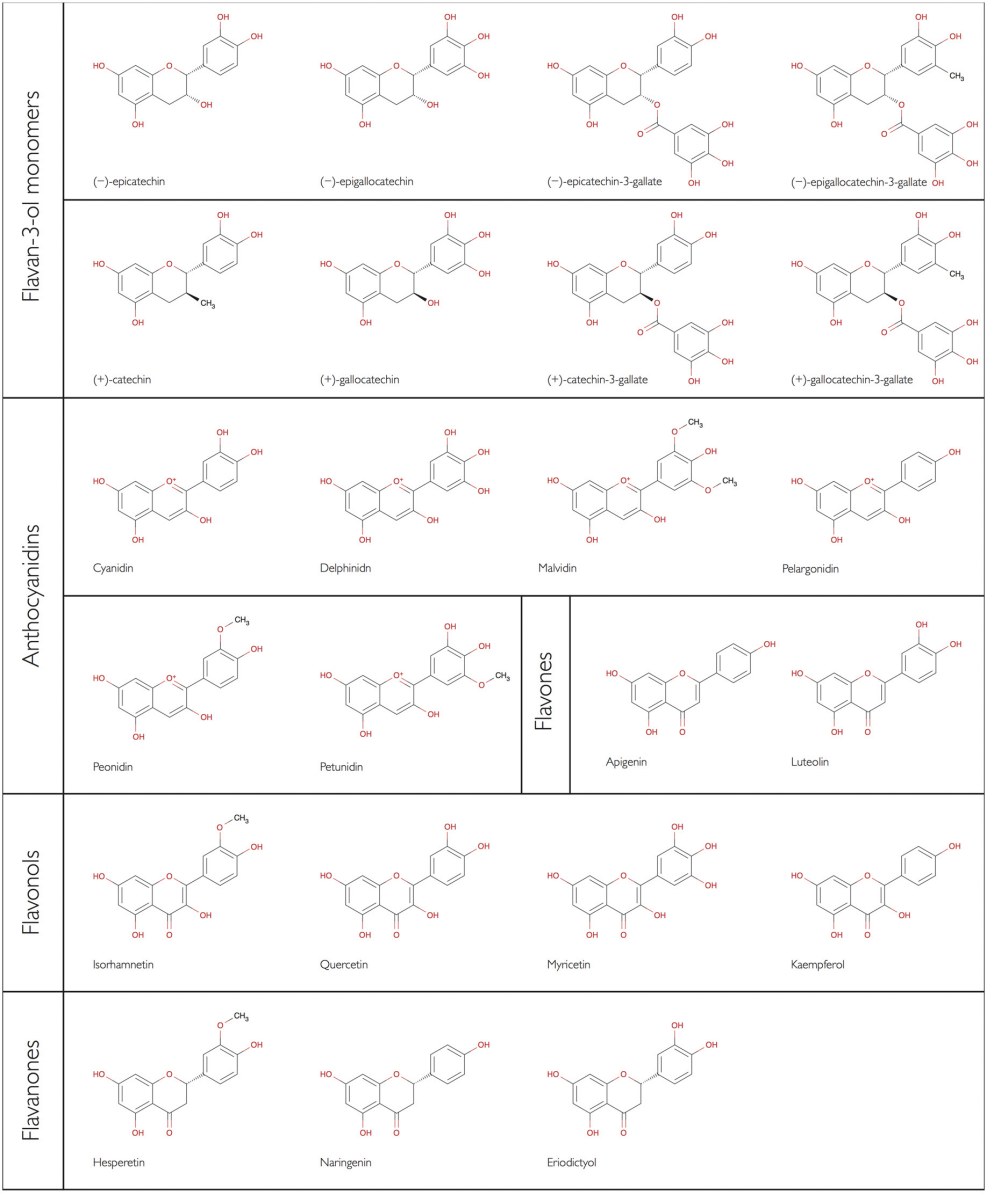
Structures of monomeric flavonoids assessed in this study.

The development of dietary recommendations and also the interpretation of results from intervention studies, requires reliable information on habitual flavonoid intake in the general public. This information is also important to assess the potential health impact of these compounds. However, there is a paucity of data available. While the flavonoid consumption data of the *European Prospective Investigation into Cancer* (EPIC) provides detailed information on flavonoid intake in this cohort [34], the data are not representative of the European public because of selection bias. Previously, we have used a novel food composition database, in combination with food consumption data from the European Food Safety Authority (EFSA), to determine flavan-3-ol intake in the European Union [35]. We have now used the same methods and data to investigate the habitual consumption of selected monomeric and polymeric flavonoids (see Fig 1 for a summary of the monomeric compounds included in this study) in the adult population of the European Union.

## Subjects and Methods

We have used the methods described previously [35] to determine mean and median intake of flavonoids, using the 2011 EFSA Comprehensive European Food Consumption Database. This database contains food consumption data for adults aged 18–64 years from 21 surveys (~30,000 individuals) and 14 countries [36]. Information concerning the methodologies used in each survey is presented in Table 1. Food consumption statistics are reported in g/day and for habitual consumption. For each country, consumption data are given according to the 1^st^ (20 categories) and 2^nd^ (140 categories) level of the FoodEx system and for the total population [37]. The hierarchical food classification system ‘FoodEx’, developed by EFSA, was used to codify all food items. Food intake by non-consumers is reported as 0.

**Table 1:** Dietary surveys for adults included in the EFSA Comprehensive European Food Consumption Database.

Country	Name of the dietary survey	Survey period	No of subjects	Method	Replicates
Belgium	Diet National 2004	2004–5	1,304	24h recall	2
Czech Republic	SISP04	2003–04	1,666	24h recall	2
Denmark	Danish Dietary Survey	2000–02	2,822	Food record	7
Finland	FINDIET 2007	2007	1,575	48h recall	1
France	INCA2	2005–07	2,278	Food record	7
Germany	National Nutrition Survey II	2005–07	10,419	24h recall	2
Hungary	National Repr Surv	2003	1,074	Food record	3
Ireland	NSIFCS	1997–99	958	Food record	7
Italy	INRAN-SCAI 2005–06	2005–06	2,313	Food record	3
Latvia	EFSA_TEST	2008	1,306	24h recall	2
Netherlands	DNFCS-2003	2003	750	24h recall	2
Spain	AESAN	1999–01	410	Food record	3
	AESAN-FIAB	2009	981	24h recall	2
Sweden	RIKSMATEN 1997–98	1997–98	1,210	Food record	7
UK	NDNS	2000–01	1,724	Food record	7

See http://www.efsa.europa.eu/en/datexfoodcdb/datexfooddb.htm2011 for details.

In order to estimate the flavonoid intake in Europe, we expanded the *FLAVIOLA Flavanol Food Composition database* which contains food composition data for approximately 3,000 food items as described previously [35]. This database is based on the US Department of Agriculture database for the Flavonoid Content of Selected Foods [38], and the proanthocyanidins (PA) Content of Selected Foods [39], expanded with values from Phenol-Explorer, a comprehensive database on the polyphenol content of foods [40]. These databases are the most up-to-date databases on flavonoids and polyphenols, and include information for 500, 205, and 456 food items for flavonoids [38], PA [39] and polyphenols [40], respectively. We expanded and completed the FLAVIOLA Flavanol Food Composition database using food data underlying DINER (Data Into Nutrients for Epidemiological Research), a data-entry system for food records created for EPIC (European Prospective Investigation into Cancer and Nutrition)-Norfolk [41]. This database was used for the calculation of the estimated flavonoid content of ~2,500 food items (82% of the 3000 total foods), using approximately 800 recipes. Retention and cooking factors for approximately 500 food items were used to estimate the effect of processing (e.g. frying, boiling, roasting, etc.) [42–44]. When information on the ingredients of commercial food products was provided by manufacturers, their flavonoid content was also calculated; where no information was available, products were matched to similar ones if possible. Food items thought not to contain significant levels of flavanols (fish, meat and eggs) were treated as logical zeros and omitted from the database. Dietary intake was estimated for the following major flavonoid subgroups (see also Fig 1 and Table 2): flavan-3-ol monomers ((–)-epicatechin, (–)-epicatechin-3-gallate, (–)-epigallocatechin. (–)-epigallocatechin-3-gallate, (+)-catechin, (+)-gallocatechin, (+)-catechin-3-gallate, (+)-gallocatechin-3-gallate), theaflavins (theaflavin, thearubigins, theaflavin-3,3'-digallate, theaflavin-3'-gallate, theaflavin-3-gallate), proanthocyanidins [PA] (dimers, trimers, 4-6mers, 7-10mers, >10mers), anthocyanidins (cyanidin, delphinidin, malvidin, pelargonidin, peonidin, petunidin), flavones (apigenin, luteolin), flavonols (isorhamnetin, quercetin, myricetin, kaempferol) and flavanones (hesperetin, naringenin, eriodictyol). Isoflavonoids, a subgroup of flavonoids with oestrogenic activity [45], are not included in this study.

**Table 2:** Names and synonyms of monomeric flavonoids used.

Compound	Synonyms
Flavan-3-ols	
(–)-epicatechin	L-epicatechin, (–)-epicatechol, L-acacatechin
(–)-epigallocatechin	gallocatechol, epigallocatechol,
(–)-epicatechin-3-gallate	
(–)-epigallocatechin-3-gallate	EGCG
(+)-catechin	catechinic acid, catechuic acid, **catergen**, (+)-cianidanol, (+)-cyanidanol, (+)-cyanidanol-3
(+)-gallocatechin	gallocatechol
(+)-catechin-3-gallate	
(+)-gallocatechin-3-gallate	gallocatechol-gallate
Anthocyanidins	
Cyanidin	cyanidol, **gastrotelos**
Delphinidin	delphinidol, ephdine
Malvidin	
Pelargonidin	pelargonidol
Peonidin	
Petunidin	petunidol
Flavones	
Apigenin	spigenin, versulin, apigenol
Luteolin	luteolol, flacitran, digitoflavone
Flavonols	
Isorhamnetin	isorhamnetol, 3'-methyl-quercetin
Quercetin	sophoretin, meletin, xanthaurine, quercetol, quercetin, quertine, flavin meletin
Myricetin	cannabiscetin, myricetol
Kaempferol	kaempferol, populnetin, rhamnolutein, robigenin, trifolitin, pelargidenolon, rhamnolutin, swartziol
Flavanones	
Hesperetin	hesperitin, hesperin, 4'-methyl-cyanidanon
Naringenin	
Eriodictyol	

We present estimated mean and median values of flavonoid intake in mg/d. Further tables are based on mean intake values to allow for comparison with other studies. The European countries represented in the EFSA comprehensive database were grouped in regions to allow for observations in each distinctive area (South: Italy, Spain, France; Central: Belgium, Czech Republic, Germany, Hungary, Ireland, Latvia, Netherlands, UK; North: Denmark, Finland, Sweden). Unless indicated otherwise, all data are given as the mean daily intake of the respective flavonoid.

### Availability of data

The flavonoid consumption data used in this study is available as S1 File: Flavonoid intake in adults in the European Union by country and individual food.

### Data processing and data analysis

The data available do not allow any statistical analyses, for example a comparison between different countries. Means shown are not weighted. Data analyses were conducted with R 3.1 [46].

## Results

### Overall intake

**Fig 2:**
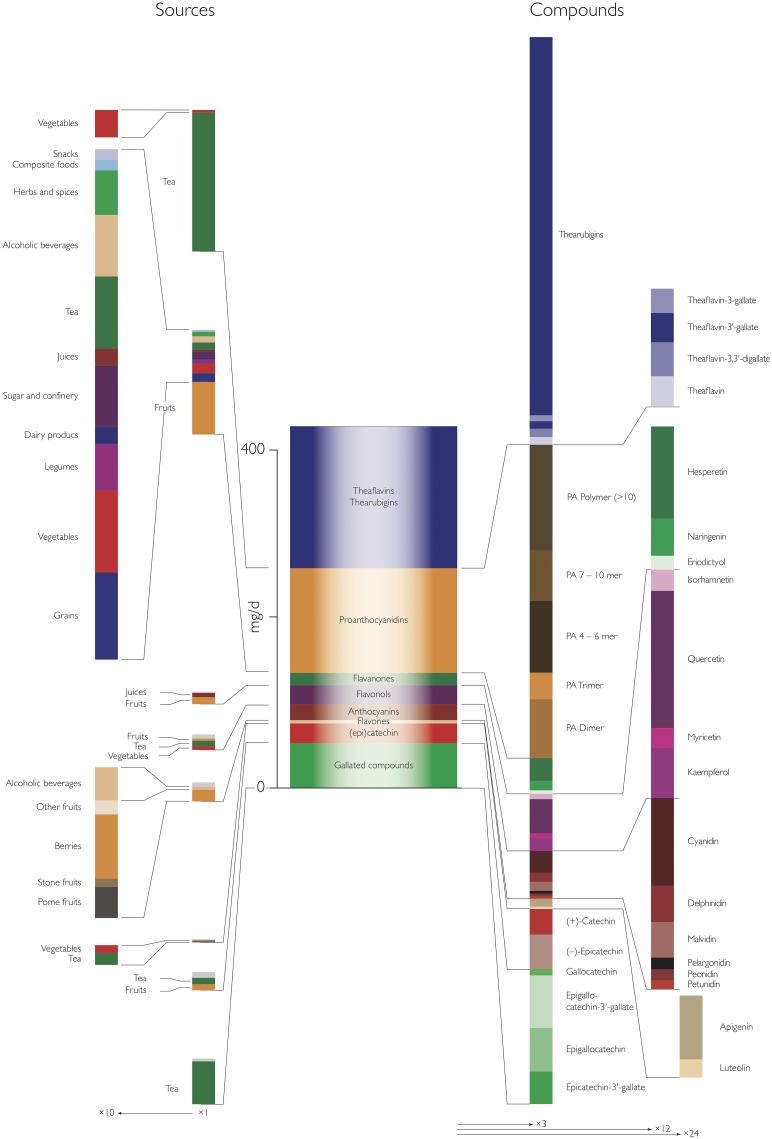
Main types and sources of flavonoids consumed by adults (18 to 64 years) in the European Union. Data is based on mean flavonoid intake of all countries included in the survey.

Data on flavonoid intake in adults was available for fourteen European countries, based on surveys of more than 30,000 participants (Table 1). All studies were conducted between 1999 and 2005. Table 3 and Fig 2 show a summary of the mean and median intake of flavonoids and flavonoid sub-groups. Mean intake of total flavonoids, including oligomeric compounds, was 428 mg/d with the highest intake in the Central Region (506 mg/d), followed by the Northern Region (348 mg/d) and the Southern Region (301 mg/d). The main types of flavonoids consumed were thearubigins and theaflavins (168 mg/d), mainly from tea, and oligo- and polymeric proanthocyanidins (124 mg/d), mainly from fruits and vegetable.

**Table 3:** Mean and median flavonoid intake (mg/d) of adults (18 to 64 years) in the European Union by country and region (mean ± SEM).

	(Epi)catechin		Gallated compounds^1^		Anthocyanidins^2^		Flavonols^3^		Flavanones^4^		Flavones^5^		Flavonoids (monomeric compounds only)^6^		Proanthocyanidins^7^		Theaflavins and Thearubigins^8^		Flavonoids (including polymers)	
	Mean	Median	Mean	Median	Mean	Median	Mean	Median	Mean	Median	Mean	Median	Mean	Median	Mean	Median	Mean	Median	Mean	Median
Denmark	25	8	43	2	25	9	19	6	13	3	3	1	128	28	116	39	135	0	379	67
Finland	22	2	34	1	28	2	17	2	30	3	3	1	134	10	115	3	105	0	354	13
Sweden	20	6	32	8	19	5	18	7	19	3	2	1	110	30	100	34	99	24	310	88
Northern Region	22 ± 1	5 ± 2	36 ± 3	4 ± 2	24 ± 3	5 ± 2	18 ± 1	5 ± 1	21 ± 5	3 ± 0	3 ± 0	1 ± 0	124 ± 7	23 ± 6	111 ± 5	26 ± 11	113 ± 11	8 ± 8	348 ± 20	56 ± 22
Belgium	20	1	25	0	19	0	19	1	12	0	3	0	97	3	109	4	75	0	281	8
Czech Republic	17	4	14	11	14	1	16	5	10	0	4	2	75	24	107	21	43	33	225	78
Germany	33	1	79	0	22	0	27	1	19	0	3	0	181	3	143	5	249	0	573	7
Hungary	22	10	47	29	15	5	23	10	11	0	4	2	122	56	141	65	145	83	408	204
Ireland	34	25	156	150	9	2	38	28	8	0	3	1	249	206	98	46	505	481	851	733
Latvia	20	6	52	39	24	1	25	13	4	0	9	2	135	61	113	13	164	125	411	199
Netherlands	31	11	108	68	11	0	31	11	18	1	2	0	201	92	96	20	347	222	643	334
United Kingdom	30	15	110	85	16	2	28	17	9	0	2	1	195	119	109	37	352	271	655	427
Central Region	26 ± 2	9 ± 3	74 ± 17	48 ± 18	16 ± 2	1 ± 1	26 ± 2	11 ± 3	11 ± 2	0 ± 0	4 ± 1	1 ± 0	157 ± 21	70 ± 24	114 ± 6	26 ± 8	235 ± 56	152 ± 59	506 ± 75	249 ± 87
France	22	3	31	1	28	3	18	4	10	0	7	3	115	14	144	20	93	0	352	34
Italy	17	4	12	1	17	4	20	7	20	1	10	6	96	22	161	35	34	0	291	57
Spain	19	4	5	2	17	4	15	5	17	0	3	1	75	16	178	33	6	0	260	48
Southern Region	19 ± 2	4 ± 0	16 ± 8	1 ± 0	20 ± 4	4 ± 0	18 ± 1	5 ± 1	15 ± 3	1 ± 0	7 ± 2	3 ± 1	95 ± 11	17 ± 2	161 ± 10	29 ± 5	44 ± 26	0 ± 0	301 ± 27	47 ± 7
Europe	24 ± 2	7 ± 2	53 ± 12	28 ± 12	19 ± 2	3 ± 1	23 ± 2	8 ± 2	14 ± 2	1 ± 0	4 ± 1	1 ± 0	136 ± 14	49 ± 15	124 ± 7	27 ± 5	168 ± 39	89 ± 38	428 ± 49	164 ± 55

^1^) epigallocatechin, epicatechin-3-gallate, epigallocatechin-3-gallate, catechin-3-gallate, gallocatechin, gallocatehin-3-gallate;

^2^) cyanidin, delphinidin, malvidin, pelargonidin, peonidin, petunidin;

^3^) Isorhamnetin, quercetin, myricetin, kaempferol;

^4^) hesperetin, naringenin, eriodictyol;

^5^) apigenin, luteolin;

^6^) sum of (epi)catechin, gallated compounds, anthocyanidins, flavonols, flavanones and flavones;

^7^) degree of polymerization >2;

^8^) theaflavin, theaflavin-3-gallate, theaflavin-3'-gallate, theaflavin-3, 3'-digallate, thearubigins

The highest intake of monomeric flavonoids was also found in the Central Region (157 mg/d), followed by the Northern (124 mg/d) and Southern (95 mg/d) Region. The distribution of intake was skewed for all compounds with a considerably lower median intake for all flavonoid classes. The main type of monomeric compounds consumed were flavan-3-ols, of which 53 mg/d were gallated and 24 mg/d non-gallated compounds.

### Regional variation

**Fig 3:**
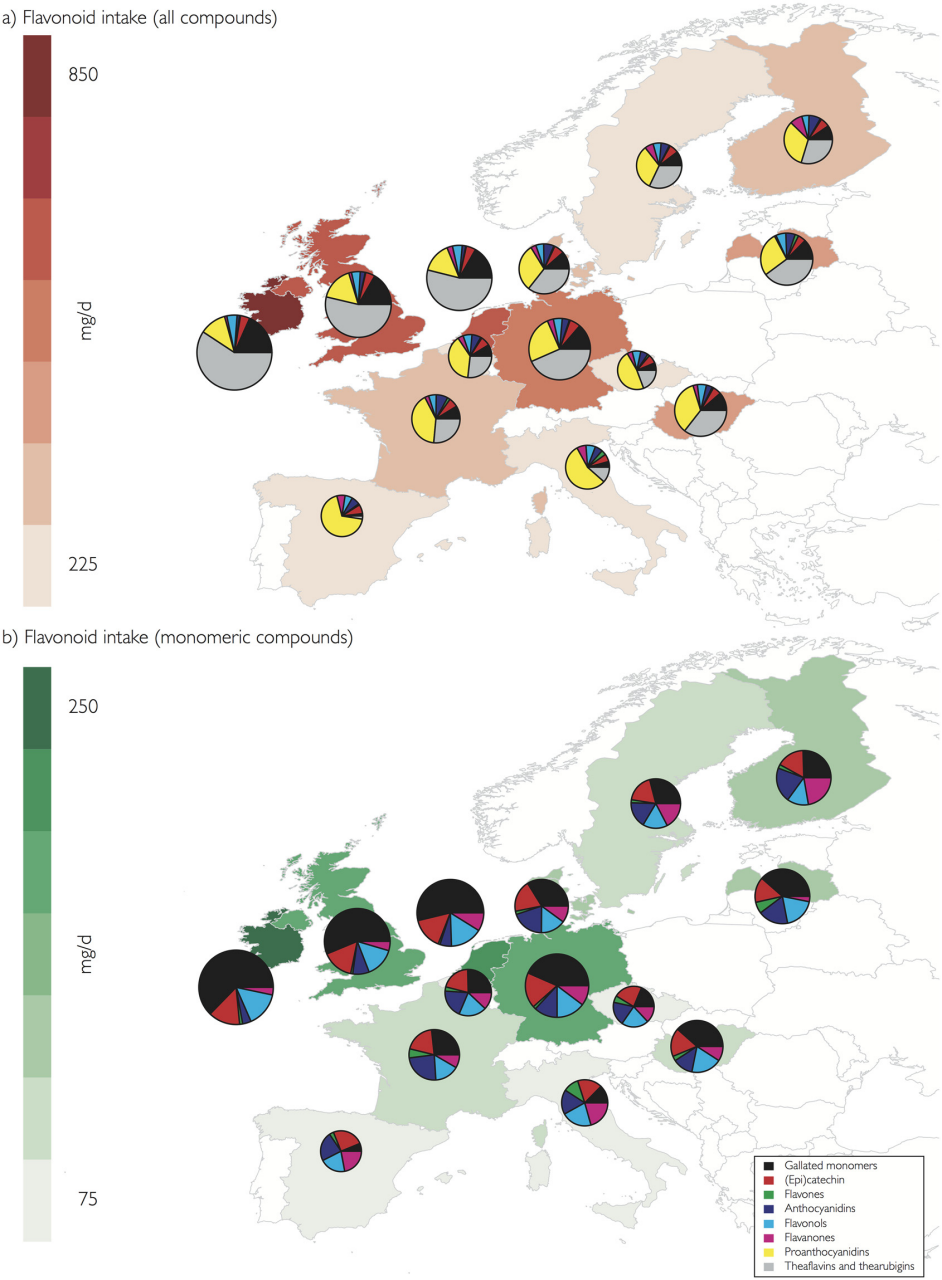
Flavonoid intake of adults (18 to 64 years) in the European Union. The maps show mean flavonoid intake in mg/d per country, the pie charts the relative contribution of different types of flavonoids.

There were noticeable differences in flavonoid intake between different countries and European regions (Fig 3). This was in particular pronounced for theaflavins and thearubigins where mean intake in the Central Region (235 mg/d) was more than double than intake in the Northern region (113 mg/d) and five times higher than in the Southern Region (44 mg/d). Moreover, even in the Central Region we observed a wide range of intake, with low intakes in the Czech Republic (43 mg/d) and Belgium (75 mg/d) and high intake in Ireland (505 mg/d), the UK (352 mg/d) and the Netherlands (347 mg/d). Conversely, intake of proanthocyanidins was highest in the Southern Region (161 mg/d) and similar in the Central (114 mg/d) and Northern (111 mg/d) Region. For all other compound classes, intake was highest in the Central Region, except for flavones and anthocyanidins, for which the highest intake was observed in the Northern Region, in particular in Finland.

### Flavonoid sources

Fig 2 shows a summary of the overall main contributors to total flavonoid intake and regional data are shown in Tables 4 and 5. Non-alcoholic beverages, in particular tea, are the main source of total and monomeric flavonoids in the European Union (mean: 66 mg/d) with noticeable difference between countries and regions. In the Northern and Central region, flavonoids from non-alcoholic beverages are the main contributors, but in the Southern region the main contributors are fruits and fruit products. Most other food sources contributed only little to overall intake.

**Table 4:** Sources of monomeric flavonoids (in mg/d of total flavonoids; mean ± SEM) of adults (18 to 64 years) in the European Union by country and region.

	Total intake Grains and grain-based products		Vegetables and vegetable products (including fungi)		Starchy roots and tubers		Legumes, nuts and oilseeds		Fruit and fruit products		Milk and dairy products		Sugar and confectionary		Fruit and vegetable juices		Non-alcoholic beverages (except milk based beverages)		Alcoholic beverages		Herbs, spices and condiments		Composite food (including frozen products)		Snacks, desserts, and other foods	
	Mean	Median	Mean	Median	Mean	Median	Mean	Median	Mean	Median	Mean	Median	Mean	Median	Mean	Median	Mean	Median	Mean	Median	Mean	Median	Mean	Median	Mean	Median
Denmark	1	0	8	5	1	1	0	0	30	7	2	1	2	1	12	3	55	1	16	9	1	0	0	0	0	0
Finland	0	0	7	2	1	1	1	0	56	0	3	2	1	0	15	4	43	1	4	0	2	0	1	0	0	0
Sweden	3	2	2	1	1	1	0	0	29	5	2	1	1	0	14	4	41	10	7	0	1	0	8	4	1	1
Northern Region	1 ± 1	1 ± 1	6 ± 2	3 ± 1	1 ± 0	1 ± 0	0 ± 0	0 ± 0	39 ± 9	4 ± 2	2 ± 0	1 ± 0	1 ± 0	0 ± 0	13 ± 1	4 ± 0	46 ± 4	4 ± 3	9 ± 4	3 ± 3	1 ± 0	0 ± 0	3 ± 2	1 ± 1	1 ± 0	0 ± 0
Belgium	2	1	6	1	1	1	1	0	27	0	2	0	2	0	6	0	31	1	11	0	1	0	6	0	1	0
Czech Republic	2	1	25	16	1	1	1	0	27	3	1	0	1	0	3	0	0	0	9	0	5	2	1	0	0	0
Germany	2	1	6	0	1	0	1	0	34	0	1	0	2	0	20	0	100	1	8	0	2	0	4	0	1	0
Hungary	1	0	10	4	1	1	1	0	35	15	1	0	1	0	5	0	57	33	3	0	4	3	0	0	0	0
Ireland	2	1	10	7	3	2	1	0	17	3	1	0	2	1	3	0	200	191	7	0	3	1	1	0	1	0
Latvia	2	1	2	1	1	1	0	0	34	0	1	0	1	0	4	0	66	50	1	0	8	0	13	8	1	0
Netherlands	2	1	8	0	1	1	1	0	19	0	3	0	2	1	14	0	139	89	5	0	1	0	5	0	2	0
United Kingdom	2	2	8	5	1	1	2	1	19	2	1	0	2	1	7	0	140	108	10	0	2	0	0	0	1	0
Central Region	2 ± 0	1 ± 0	9 ± 2	4 ± 2	1 ± 0	1 ± 0	1 ± 0	0 ± 0	27 ± 3	3 ± 2	1 ± 0	0 ± 0	1 ± 0	0 ± 0	8 ± 2	0 ± 0	92 ± 23	59 ± 24	7 ± 1	0 ± 0	3 ± 1	1 ± 0	4 ± 2	1 ± 1	1 ± 0	0 ± 0
France	2	2	8	3	1	1	2	1	31	1	2	1	1	0	10	0	38	0	12	2	7	2	0	0	1	1
Italy	1	1	10	5	1	0	1	0	41	10	1	0	0	0	3	0	13	0	11	0	14	5	0	0	1	0
Spain	2	1	15	5	1	1	2	0	39	7	2	1	1	0	5	0	1	0	5	0	2	1	0	0	1	0
Southern Region	2 ± 0	1 ± 0	11 ± 2	4 ± 0	1 ± 0	1 ± 0	1 ± 0	0 ± 0	37 ± 3	6 ± 2	1 ± 0	1 ± 0	1 ± 0	0 ± 0	6 ± 2	0 ± 0	17 ± 11	0 ± 0	9 ± 2	1 ± 1	8 ± 3	3 ± 1	0 ± 0	0 ± 0	1 ± 0	0 ± 0
Europe	2 ± 0	1 ± 0	9 ± 1	4 ± 1	1 ± 0	1 ± 0	1 ± 0	0 ± 0	31 ± 3	4 ± 1	2 ± 0	1 ± 0	1 ± 0	0 ± 0	8 ± 1	1 ± 0	66 ± 16	35 ± 15	8 ± 1	1 ± 1	4 ± 1	1 ± 0	3 ± 1	1 ± 1	1 ± 0	0 ± 0

Meat, meat products, fish and other sea-foods did not contribute to total flavonoid intake and were omitted.

**Table 5:** Sources of proanthocyanidins (in mg/d of proanthocyanidins with a degree of polymerisation of two or more; mean ± SEM) of adults (18 to 64 years) in the European Union by country and region.

	Total intake Grains and grain-based products		Vegetables and vegetable products (including fungi)		Legumes, nuts and oilseeds		Fruit and fruit products		Milk and dairy products		Sugar and confectionary		Fruit and vegetable juices		Non-alcoholic beverages (excepting milk based beverages)		Alcoholic beverages		Herbs, spices and condiments		Composite food (including frozen products)		Snacks, desserts, and other foods	
	Mean	Median	Mean	Median	Mean	Median	Mean	Median	Mean	Median	Mean	Median	Mean	Median	Mean	Median	Mean	Median	Mean	Median	Mean	Median	Mean	Median
Denmark	4	1	4	0	2	0	68	23	3	0	9	4	3	1	6	0	16	9	0	0	0	0	1	0
Finland	4	0	15	0	4	0	69	0	3	2	7	0	4	1	5	0	3	0	0	0	0	0	1	0
Sweden	20	13	0	0	3	0	46	15	2	1	7	0	2	1	7	1	7	0	0	0	5	3	1	1
Northern Region	9 ± 6	5 ± 4	6 ± 4	0 ± 0	3 ± 1	0 ± 0	61 ± 7	13 ± 7	3 ± 0	1 ± 0	8 ± 1	1 ± 1	3 ± 1	1 ± 0	6 ± 1	0 ± 0	9 ± 4	3 ± 3	0 ± 0	0 ± 0	2 ± 2	1 ± 1	1 ± 0	0 ± 0
Belgium	10	4	8	0	3	0	52	0	2	0	11	0	2	0	5	0	11	0	2	0	1	0	1	0
Czech Republic	11	6	11	0	5	0	59	11	1	0	3	0	1	0	0	0	7	0	7	4	1	0	1	0
Germany	12	4	6	0	7	0	80	0	1	0	9	0	6	0	13	0	7	0	0	0	0	0	1	0
Hungary	5	1	16	0	7	0	86	53	3	0	4	0	1	0	7	4	3	0	8	7	0	0	0	0
Ireland	10	7	7	0	3	0	33	10	1	0	10	5	1	0	24	23	5	0	3	1	0	0	1	0
Latvia	11	3	0	0	3	0	70	0	1	0	6	0	1	0	8	6	1	0	1	0	10	4	1	0
Netherlands	10	5	5	0	5	0	30	0	4	0	13	4	3	0	17	11	4	0	0	0	1	0	3	0
United Kingdom	18	11	0	0	13	6	38	4	1	0	8	3	2	0	17	13	9	0	2	0	0	0	1	0
Central Region	11 ± 1	5 ± 1	7 ± 2	0 ± 0	6 ± 1	1 ± 1	56 ± 8	10 ± 6	2 ± 0	0 ± 0	8 ± 1	2 ± 1	2 ± 1	0 ± 0	11 ± 3	7 ± 3	6 ± 1	0 ± 0	3 ± 1	1 ± 1	2 ± 1	0 ± 0	1 ± 0	0 ± 0
France	14	10	19	0	8	1	70	5	2	1	7	0	2	0	6	0	12	2	4	0	0	0	2	1
Italy	8	3	9	0	3	0	83	32	1	0	2	0	1	0	2	0	11	0	41	0	0	0	1	0
Spain	11	5	52	0	11	0	85	23	2	1	4	0	1	0	0	0	5	0	5	2	0	0	1	0
Southern Region	11 ± 2	6 ± 2	27 ± 13	0 ± 0	7 ± 2	0 ± 0	80 ± 5	20 ± 8	1 ± 0	1 ± 0	4 ± 1	0 ± 0	1 ± 0	0 ± 0	2 ± 2	0 ± 0	9 ± 2	1 ± 1	16 ± 12	1 ± 1	0 ± 0	0 ± 0	2 ± 0	0 ± 0
Europe	10 ± 1	5 ± 1	11 ± 4	0 ± 0	5 ± 1	0 ± 0	62 ± 5	13 ± 4	2 ± 0	0 ± 0	7 ± 1	1 ± 1	2 ± 0	0 ± 0	8 ± 2	4 ± 2	7 ± 1	1 ± 1	5 ± 3	1 ± 1	1 ± 1	0 ± 0	1 ± 0	0 ± 0

Starchy roots and tubers, meat, meat products, fish and other sea-foods did not contribute to total flavonoid intake and were omitted.

**Fig 4:**
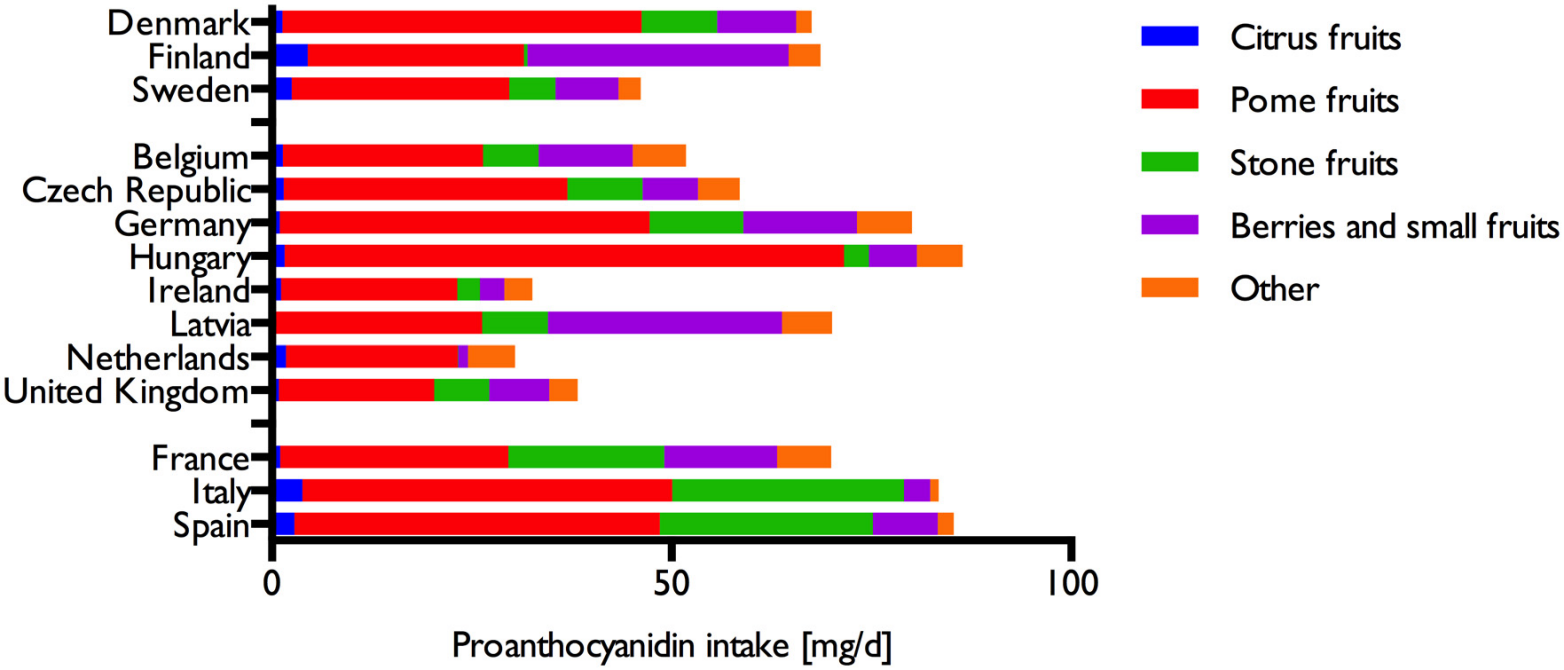
Contribution of different fruits to total proanthocyanidins.

**Fig 5:**
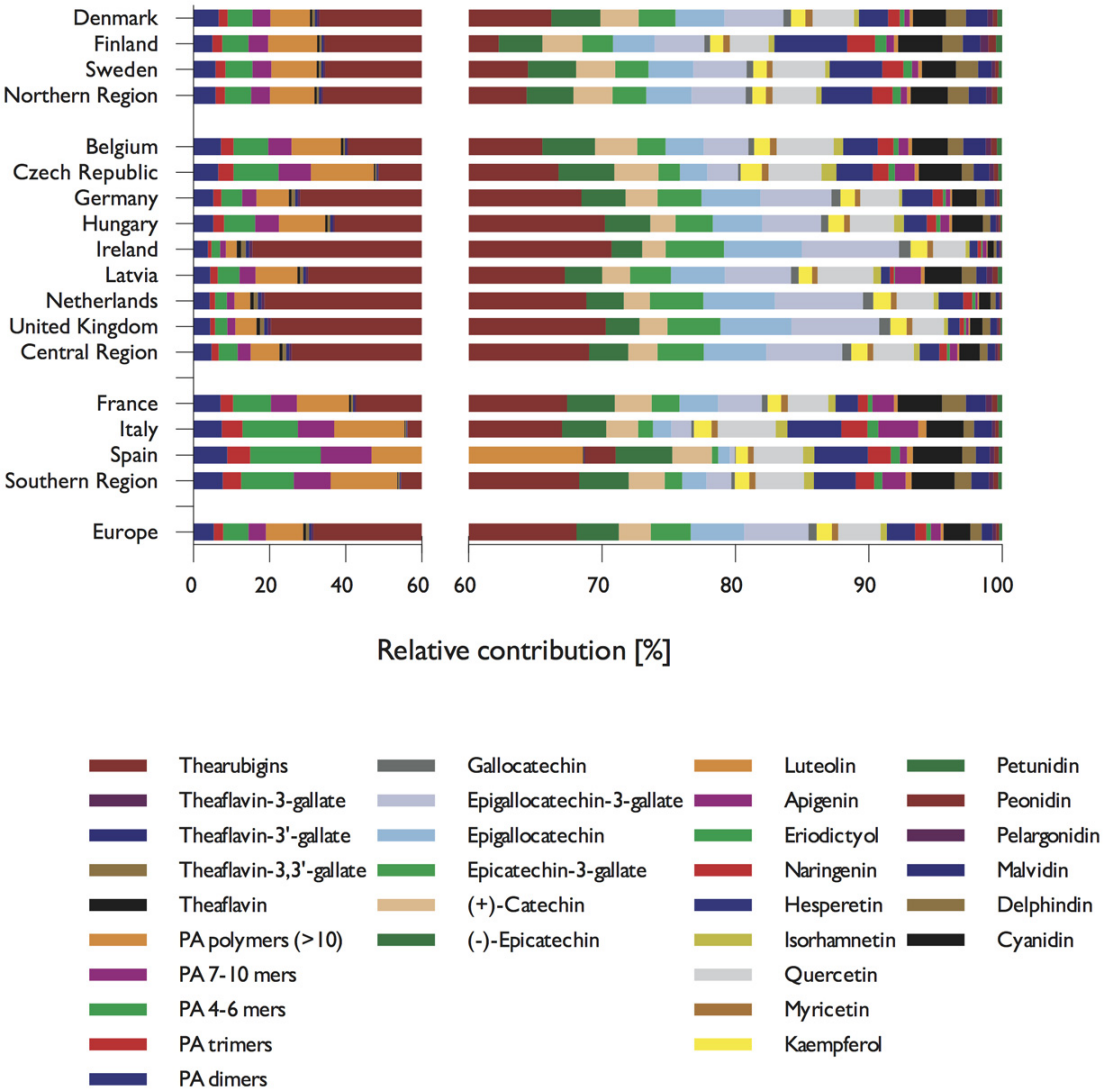
Relative contribution of individual compounds to total flavonoid intake.

Whereas tea was the main contributor to dietary theaflavins and thearubigins, there was a wider variety of sources for proanthocyanidins (Table 5, Fig 4). The main sources were fruits (mean: 62 mg/d), in particular pome fruits (35 mg/d), berries and small fruits (11 mg/d) and stone fruits (10 mg/d). There were however large variations between different countries (Fig 5): in Finland and Latvia, berries and other small fruits were a major contributor to proanthocyanidins intake, whereas in the Southern Region, in particular in Italy and Spain, stone fruits were an important source. In comparison to polymeric and monomeric flavan-3-ols, the contribution from other groups were small and their main sources were fruits.

### Individual compounds and sources

**Fig 6:**
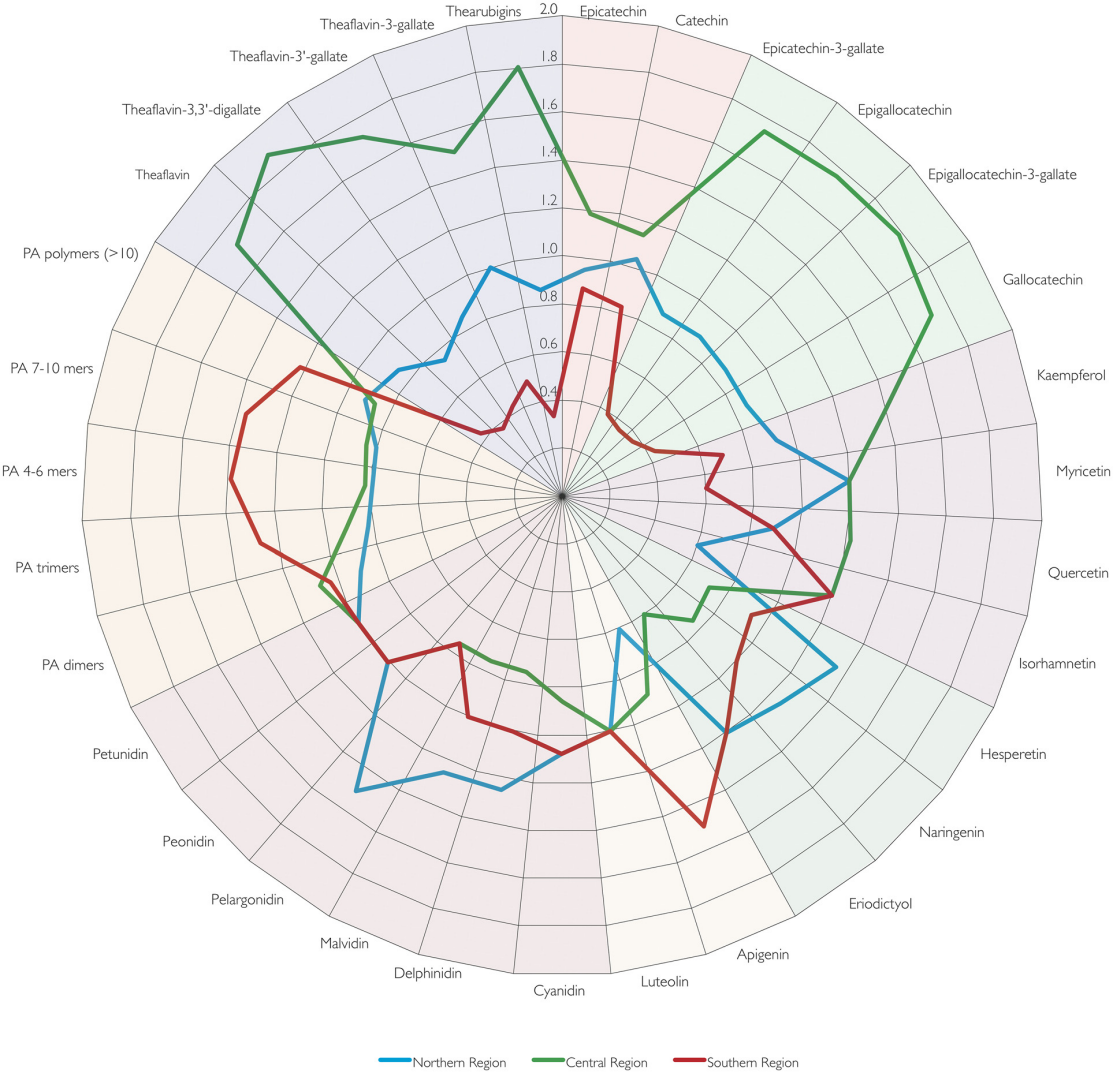
Comparison of relative intake of individual flavonoids by region. The graph shows the ratio of average intake by region and average intake in Europe.

The contribution of different compounds to total flavonoid intake is summarised in Fig 2, and shown by region in Table 6 and Fig 6. Thearubigins were the largest contributor to total flavonoid intake (156 mg/d) whereas theaflavins contributed only small amounts, and this can be observed in all countries, independent of the total amount consumed. Conversely, the composition of proanthocyanidins is more variable. Overall, polymeric proanthocyanidins (DP > 10) contribute most to total intake, but the contribution of different oligomers is very variable. Especially in countries of the Southern Region, intake of 4-6mers is much higher than in most other countries (Fig 3).

**Table 6:** Mean intake of individual compounds (mg/d ± SEM) in adults (18 to 64 years) in the European Union.

	Denmark	Finland	Sweden	Northern Region	Belgium	Czech Republic	Germany	Hungary	Ireland	Latvia	Netherlands	United Kingdom	Central Region	France	Italy	Spain	Southern Region	Europe
(–)-Epicatechin	14	12	11	12 ± 1	11	9	19	14	20	12	18	17	15 ± 1	13	10	11	11 ± 1	14 ± 1
(+)-Catechin	11	11	9	10 ± 1	9	7	14	8	15	9	13	14	11 ± 1	10	7	8	8 ± 1	10 ± 1
(–)-Epicatechin-3-gallate	10	8	8	9 ± 1	6	4	19	12	37	13	26	26	18 ± 4	7	3	1	4 ± 2	13 ± 3
(–)-Epigallocatechin	14	11	10	12 ± 1	8	5	25	15	50	17	34	35	24 ± 5	10	4	2	5 ± 2	17 ± 4
(–)-Epigallocatechin-3-gallate	17	13	12	14 ± 1	9	5	31	18	62	20	42	43	29 ± 7	12	4	1	6 ± 3	21 ± 5
(+)-Gallocatechin	2	2	2	2 ± 0	1	1	4	2	8	2	5	5	4 ± 1	2	1	0	1 ± 0	3 ± 1
Kaempferol	4	3	3	4 ± 0	3	4	6	5	11	4	8	8	6 ± 1	3	4	2	3 ± 0	5 ± 1
Myricetin	2	2	1	2 ± 0	1	1	2	2	4	2	3	3	2 ± 0	2	1	1	1 ± 0	2 ± 0
Quercetin	12	10	12	11 ± 1	12	9	17	14	21	17	18	16	15 ± 1	11	13	10	11 ± 1	14 ± 1
Isorhamnetin	1	2	1	1 ± 0	2	3	2	3	3	2	2	2	2 ± 0	2	3	2	2 ± 0	2 ± 0
Hesperetin	8	19	12	13 ± 3	7	6	13	7	5	3	12	6	7 ± 1	6	12	10	9 ± 2	9 ± 1
Naringenin	3	7	5	5 ± 1	3	3	4	3	2	1	4	2	3 ± 0	3	6	4	4 ± 1	4 ± 0
Eriodictyol	1	3	2	2 ± 1	1	1	1	1	1	0	2	1	1 ± 0	1	2	2	2 ± 0	1 ± 0
Apigenin	2	2	1	2 ± 0	2	3	2	3	3	8	1	1	3 ± 1	6	9	1	5 ± 2	3 ± 1
Luteolin	1	1	1	1 ± 0	1	1	1	1	1	1	1	1	1 ± 0	1	2	1	1 ± 0	1 ± 0
Cyanidin	9	12	8	10 ± 1	8	7	11	9	4	12	6	6	8 ± 1	12	8	10	10 ± 1	9 ± 1
Delphinidin	6	5	5	5 ± 0	3	2	3	2	2	4	2	4	3 ± 0	6	2	3	4 ± 1	4 ± 0
Malvidin	6	5	3	5 ± 1	5	3	4	2	2	3	2	3	3 ± 0	5	4	3	4 ± 1	3 ± 0
Pelargonidin	2	2	1	2 ± 0	1	1	1	1	1	2	1	1	1 ± 0	2	1	1	1 ± 0	1 ± 0
Peonidin	1	2	1	1 ± 0	1	1	1	1	0	2	0	1	1 ± 0	1	1	1	1 ± 0	1 ± 0
Petunidin	1	2	1	1 ± 0	1	1	1	0	0	1	0	1	1 ± 0	1	1	1	1 ± 0	1 ± 0
PA dimers	25	18	18	20 ± 2	20	15	30	22	33	18	27	29	24 ± 2	25	22	23	23 ± 1	23 ± 1
PA trimers	9	9	8	9 ± 0	9	9	12	12	9	8	9	9	10 ± 1	12	16	16	14 ± 1	10 ± 1
PA 4–6 mers	25	25	22	24 ± 1	26	27	31	33	19	23	20	21	25 ± 2	35	42	48	42 ± 4	28 ± 2
PA 7–10 mers	18	18	15	17 ± 1	17	19	22	25	13	17	13	14	18 ± 2	24	28	35	29 ± 3	20 ± 2
PA polymers (>10)	39	45	37	40 ± 3	37	37	48	50	25	45	26	36	38 ± 3	48	54	56	53 ± 2	42 ± 3
Theaflavin	2	2	2	2 ± 0	1	1	4	3	9	3	6	6	4 ± 1	2	1	0	1 ± 0	3 ± 1
Theaflavin-3,3'-digallate	3	2	2	2 ± 0	2	1	5	3	10	3	7	7	5 ± 1	2	1	0	1 ± 1	3 ± 1
Theaflavin-3'-gallate	2	2	2	2 ± 0	1	1	4	3	9	3	6	6	4 ± 1	2	1	0	1 ± 0	3 ± 1
Theaflavin-3-gallate	2	2	1	2 ± 0	1	1	4	2	7	2	5	5	3 ± 1	1	0	0	1 ± 0	2 ± 1
Thearubigins	126	98	93	105 ± 10	70	40	231	135	470	152	323	327	219 ± 52	87	31	6	41 ± 24	156 ± 36

Epigallocatechin-3-gallate (EGCG) is the main type of gallated compounds consumed (21 mg/d) and (–)-epicatechin the main non-gallated monomeric flavan-3-ol. The consumption of all other types of flavonoids, except for quercetin (14 mg/d), was much lower.

### Distribution of intake

The data available did not provide much information about the distribution of intake except for median and mean values and these data allows only a limited assessment. The distribution of intake was skewed for most compounds and countries, as median intake was always lower than mean intake. This was in particular noticeable in Germany were mean monomeric flavonoid intake was more than 60-fold higher than median intake (181 mg/d *vs* 3 mg/d) and more than 80-fold higher when including polymeric compounds (523 mg/d *vs* 7 mg/d). It was noticeable that for some compounds, i.e. gallated flavan-3-ols, anthocyanidins, flavanones and flavones as well as theaflavins and thearubigins, median intake in Germany was 0 mg/d. A similar, although less extreme, distribution was observed in Belgium (97 mg/d *vs* 3 mg/d for monomeric flavonoids and 281 mg/d vs 8 mg/d for all flavonoids). Conversely, mean and median intake were very similar both in Ireland, both for monomeric compounds (249 mg/d *vs* 206 mg/d) and total flavonoids (851 mg/d *vs* 733 mg/d). The distribution of intake was most skewed for flavanones, were median intake was 0 mg/d in most countries.

## Discussion

There has been extensive research on the beneficial and adverse health effects of flavonoids, but there is only limited information on flavonoid intake in the general public. In this study, we provide one of the most comprehensive analyses of flavonoid intake in Europe, based on representative dietary surveys. The key strengths of this study are the data used, in particular the European food consumption data [36] which provides representative dietary data, and the detailed food composition data of the FLAVIOLA Flavanol Food Composition Database [35]. These data allow a reliable estimate of intake in the adult population. There are also some methodological limitations which we have discussed in more detail previously [35]. The main limitations were the resolution of data available and differences in the dietary assessment methods between countries (Table 1), which is likely to have introduced bias. In particular differences in assessment methods, such as differences in the number of days of assessment and seasonality, will affect the results. In addition, all observational methods are prone to underreporting and it is therefore likely that the actual intake of flavonoids is higher than reported here. Furthermore, the data available did not allow further statistical analyses.

More detailed data on the distribution of intake, or even the actual food consumption data such as provided by the UK’s National Diet and Nutrition Survey (NDNS) or National Health and Nutrition Examination Survey (NHANES) in the US, would have allowed a better exploration of the data available. However, despite these limitations this study, in particular in combination with our previous research [35], provides the most detailed and accurate analysis of flavonoid intake in the European Union.

### Comparison with studies

There is a paucity of data on flavonoid intake in the general public and only few estimates exist.

Johannot and Somerset [47] reanalysed data from representative surveys in Denmark [48] and the Netherlands [49, 50], but these estimates are considerably lower than estimated intake in the current study and this might be due to a smaller number of food items for which data were available. While there are only limited data from representative surveys, there are more data available from other studies, such as clinical trials and observational cohorts, or food balance sheets. Beking and Vieira estimated flavonoid intake in the UK and Ireland using the latter approach [51], and their estimates are considerably higher for anthocyanidins (60 mg/d *vs* 9 mg/d for Ireland; 69 mg/d *vs* 16 mg/d for the UK) and flavanones (29 mg/d *vs* 8 mg/d for Ireland; 26 mg/d *vs* 9 mg/d for the UK), whereas their estimates for monomeric flavan-3-ols are lower (190 mg/d vs 9 mg/d for Ireland; 140 mg/d *vs* 16 mg/d for the UK). Due to differences in the methodologies used, it is difficult to explain the reason for these differences. Zamaro-Ros *et al*. conducted one of the most detailed analysis of total flavonoid intake in the different centres of the *European Prospective Investigation into Cancer (EPIC)* study [34, 52–54]. These data provided so far the most comprehensive analysis of flavonoid intake in Europe, but intakes were considerably lower in our study: flavonol (30 mg/d *vs* 23 mg/d), flavanone (35 mg/d *vs* 14 mg/d), anthocyanidins (31 mg/d *vs* 19 mg/d) and proanthocyanidin (207 mg/d *vs* 124 mg/d) intake were considerably higher than in our study. However, there are some important differences: Firstly, the participants of EPIC are not a representative selection of the general public with some cohorts contributing only women or men and the cohorts consisting of mainly healthy, middle-aged participants, and because of volunteer bias, participants are likely to have more interest in diet and health and therefore a different diet. Secondly, intake assessment in EPIC is based on dietary data collected in the 1990s, and it is likely that dietary habits have changed.

Total intake of flavonoids in Europe is comparable with intake in Australia (454 mg/d), although the consumption of anthocyanidins (3 mg/d in Australia *vs* 19 mg/d), flavanones (7 mg/d in Australia *vs* 14 mg/d) and flavones (0.5 mg/d in Australia *vs* 4 mg/d) was considerably lower and it is mainly monomeric and polymeric flavan-3-ol that is higher (422 mg/d *vs* 370 mg/d; see [35] for a more detailed discussion) [47].

Based on data from NHANES, Bai *et al*. [55] estimated dietary intake of flavonoids in US adults. Overall, intake in the US is similar to intake in Europe, although there are some notable differences: intake of flavanones (22 mg/d *vs* 14 mg/d) is higher in the US population, but intake of flavones (4 mg/d *vs* 1 mg/d) and in particular anthocyanidins (9 mg/d *vs* 19 mg/d) is considerably lower. Indeed, the intake of anthocyanidins is lower than in all European countries except for Ireland. It is more difficult to compare intake of flavan-3-ols or total flavonoids, as Bai *et al*. did not include thearubigins. However, it is noticeable that the intake of gallated compounds is lower than intake in Europe (27 mg/d *vs* 53 mg/d) whereas the intakes of (–)-epicatechin (68 mg/d *vs* 14 mg/d) and (+)-catechin (84 mg/d *vs* 10 mg/d) are much higher. This is surprising as such a pattern of intake is difficult to explain as the main sources of (–)-epicatechin and (+)-catechin also contain similar or higher amounts of gallated compounds and thus it is not clear how such high intakes can be achieved.

Table 7 shows a comparison of mean and median intake data from Europe, Australia and the USA, both from representative surveys and large cohort studies. While differences in methodology and the definition of flavonoid subclasses, in particular polymeric compounds, make a direct comparison difficult, the results are similar for most compounds. The most obvious differences, which cannot be explained by methodological differences, are the lower intake of anthocyanidins in the USA and Australia and the higher intake of flavanones in the USA.

**Table 7:** Mean and median flavonoid intake (in mg/d) in representative nutrition surveys and observational studies.

Country/Region	Flavan-3-ols	Anthocyanidins	Flavonols	Flavanones	Flavones	PA	Thearubigins & Theaflavins	Study	Assessment method^¶^
Mean intake									
Europe^†^	77	19	23	14	4	124	168	Current study	Table 1
Europe	76	31	29	5	35	198	107	EPIC^§^ [34, 52–54]	24HDR
USA^†^	182	9.2	18	22	1	98	—	NHANES [55]	24HDR
USA	79	8	15	26	1	—	—	Nurses Health Study II [56]	FFQ
Australia^†^	—	2.9	21	7	1	—	—	Australian National Nutrition Survey (NNS95) [47]	24HDR
Median intake									
Europe^†^	35	3	8	1	1	27	89	Current study	Table 1
USA	20	<1	9	40	<1	175^‡^	—	Iowa Women’s Health [12]	FFQ
USA	17	10	13	17	1	132^‡^	—	Cancer Prevention Study II [13]	FFQ

For observational studies, mean or median intake is based on the mean/median intake of quintile 3.

^†^representative surveys;

^‡^includes monomeric compounds;

^§^non-weighed average of values;

^¶^FFQ: food frequency questionnaire, 24HDR 24h dietary recall

### Regional differences and distributions

There were large regional differences, both in the type of flavonoids consumed and the distribution of intake. We have discussed differences in the consumption of flavan-3-ols, in particular gallated compounds and proanthocyanidins, in great detail previously [35], but there were also noticeable differences for other compound classes. Intakes of anthocyanidins (in particular cyanidin) and flavanones (in particular hesperetin) were highest in the Northern Region, in particular in Finland. In Finland, the main source of anthocyanidins where berries and small fruits (18 mg/d) whereas the main source of flavanones were citrus fruits (20 mg/d). Interestingly, Finland had the highest intake of flavanones from citrus fruits in Europe, followed by Italy (17 mg/d) and Sweden (11 mg/d). Within the Central Region, there was also a large variability of intake between countries. While overall flavonoid intake in Ireland was the highest in Europe, the intake of anthocyanidins was the lowest overall, and intake of flavanones was also very low.

In this and previous studies [35], we have included France in the Southern Region as dietary intake was more comparable with intake in Italy and Spain. However, there are some important differences, and the intake of flavan-3-ols and anthocyanidins in France is considerably higher than in the other countries of the Southern Region.

The distribution of intake is very skewed, with higher mean than median intake. This is in particular noticeable for compounds with high intake such as monomeric and polymeric flavan-3-ols [35], but also for other compounds. The largest difference between mean and median intake was observed in Germany where median intake for some flavan-3-ols, but also anthocyanidins, flavanones and flavones was 0 mg/d. Although regional differences in dietary intake might be an explanation for this, as the Second German National Nutrition Survey [57] shows an almost two-fold regional variation in the consumption of fruit, the main sources of monomeric flavonoids apart from tea in Germany. However, data from EPIC Potsdam and EPIC Heidelberg show similar mean intake of anthocyanidins (34 mg/d and 37 mg/d respectively) [52] and similar results are also found for flavanones (44 mg/d and 36 mg/d) and flavones (7 mg/d and 8 mg/d).

### Implications

The mean intake of flavonoids in Europe is lower than the amounts suspected to result in adverse effects [24] and it is therefore unlikely that the habitual intake is detrimental to health. However, the main focus of flavonoid research in the past decades has focused on their potentially beneficial effect. Mean intake of most flavonoids in Europe is broadly comparable with intake in observational studies conducted in the US [12–14, 56] and Europe [15, 23] benefits to health are therefore likely to be limited. However, mean intake of anthocyanidins in some countries (Finland, Latvia and France) was similar or higher than intake in the top quintile in the Nurses Health Study II, where high anthocyanidins intake was associated with a reduced risk of myocardial infarction [56]. It was also higher than in the UK twin study [58], where high anthocyanin intake was associated with improved vascular function.

Results from observational studies are ambiguous and failed to show a clear association between flavonoid intake and health, especially for cardio-vascular diseases and all-cause mortality. Studies investigating these associations did not show significant trends suggestive of a dose-response effect, and beneficial effects are mainly observed when comparing the quantiles with lowest and very low intake [13, 15]. As this pattern is observed in several observational studies with a very wide range of flavonoid intake [12, 13, 15, 56, 59, 60], it is unlikely that there is a threshold effect. A similar pattern was observed for risk-factors of cardio-vascular diseases [61]. Conversely, a statistically significant trend suggesting a dose response effect was observed for associations with type 2 diabetes in Europe [23] and the US [62].

Previously, we have shown that flavan-3-ol intake in the general public is much lower than the amounts used in most dietary intervention studies [33] and that it is unlikely that the amount consumed has a direct impact on vascular health. While there are several systematic reviews and meta-analyses for flavan-3-ols [33, 63, 64], there is a paucity of similar data for other compounds. A recent review of dietary intervention studies with berry polyphenols found that most intervention studies were conducted with anthocyanidin intakes in excess of 200 mg/d, which is much higher than the usual intake in Europe.

## Conclusion

Our results provide a detailed overview of flavonoid consumption in European adults. These results allow for a comparison of habitual intake in the general public with the amounts used in dietary intervention studies. Our data suggest that habitual intake of most compounds is likely too low to result in a reduced risk of cardio-vascular diseases, but also too low to result in adverse effects. However, a lack of systematic reviews and meta-analyses of intervention studies makes these comparisons difficult. Further research is required to investigate the potential health benefits of flavonoids, in particular the primary prevention of chronic diseases in the general public. It is in particular important to use standardised and validated analytical methods and a consistent nomenclature of compounds to ensure that results are comparable. Furthermore, the development of improved dietary assessment methods, especially using nutritional biomarkers, is important to investigate the potential health effects of flavonoids in larger observational studies.

An important result of our study was the skewed distribution of flavonoid intake, especially in Belgium and Germany. In both countries, half of the adult population consumed only negligible amounts of monomeric and polymeric flavonoids. Most observational studies conducted so far have found the highest risk of cardio-vascular diseases with very low flavonoid intake [12–14], and even though it is not clear whether this is due to dietary or other factors, it is important to investigate whether these dietary patterns have an impact on public health.

## Supporting Information

S1 File Flavonoid intake in adults in the European Union by country and individual food (CSV)
